# Epidemiological study of hemotropic mycoplasmas (hemoplasmas) in cats from central Spain

**DOI:** 10.1186/s13071-018-2740-9

**Published:** 2018-03-20

**Authors:** David Díaz-Regañón, Alejandra Villaescusa, Tania Ayllón, Fernando Rodríguez-Franco, Mercedes García-Sancho, Beatriz Agulla, Ángel Sainz

**Affiliations:** 1 Department of Animal Medicine and Surgery, College of Veterinary Medicine, Complutense University of Madrid, Avda. Puerta de Hierro s/n, 28040 Madrid, Spain; 2 Instituto Nacional de Infectología Evandro Chagas, Fiocruz, Avenida Brazil 4365, CEP 21040- 900, Rio de Janeiro, Brazil

**Keywords:** Hemotropic mycoplasmas, Hemoplasmas, Cats, Central Spain, Polymerase chain reaction

## Abstract

**Background:**

Hemotropic mycoplasmas (hemoplasmas) have been found infecting cats worldwide. However, studies about feline hemoplasma infections in Spain are scarce. Therefore, the purpose of the research was to evaluate the prevalence of feline hemotropic mycoplasmas and to characterize risk factors and clinical findings associated with these infections in a cat population from the Madrid area, Spain.

**Methods:**

Polymerase chain reaction (PCR) was employed to detect *Mycoplasma haemofelis* (Mhf)*,* “*Candidatus* Mycoplasma haemominutum” (CMhm) and “*Candidatus* Mycoplasma turicensis” (CMt) in blood samples from 456 client-owned and 138 stray cats from Madrid. In order to assess associations between these hemoplasma infections and epidemiological parameters, data regarding signalment, environment, prophylaxis measures, retrovirus status, clinical signs and laboratory findings were compiled, whenever possible.

**Results:**

DNA of feline hemoplasmas was detected from the blood of 63 out of 594 cats (10.6%), with a prevalence of 3.7% (22/594) for Mhf, 8.1% (48/594) for CMhm and 0.5% (3/594) for CMt. Stray cats had statistically higher prevalences of feline hemoplasmas (15.9%) and, specifically, of Mhf (8.7%) than client-owned cats (9 and 2.2%, respectively). A total of seven cats (1.17%) were co-infected with “*Candidatus* M. haemominutum” and *M. haemofelis*, two (0.33%) with “*Candidatus* M. haemominutum” and “*Candidatus* M. turicensis” and another one (0.17%) with *M. haemofelis* and *Candidatus* “M. turicensis”. Male gender, collection of blood during warm months and FeLV/FIV positivity status were associated with hemotropic mycoplasma infection in cats from Madrid. Additionally, within the group of client-owned cats, hemoplasma infection was associated with adult age, outdoor access, and the existence of low haematocrit, erythrocyte count and haemoglobin concentration values.

**Conclusions:**

To our knowledge, this is the first epidemiological survey of feline hemoplasmas performed in central Spain (Madrid). Our study confirms that “*Ca.* Mycoplasma haemominutum”, *Mycoplasma haemofelis* and “*Ca.* Mycoplasma turicensis” are infecting client-owned and stray cats in this region of Spain, “*Ca.* Mycoplasma haemominutum” being the most prevalent species. More studies are necessary to help understand the role of the natural infection by these species of hemoplasma in cats.

## Background

Hemotropic mycoplasmas (the so-called hemoplasmas) are small wall-less bacteria that attach to the erythrocytes [1], causing anaemia in different mammalian species, including cats. Three hemoplasma species have been typically detected in cats: *Mycoplasma haemofelis* (Mhf) [2], “*Candidatus* Mycoplasma haemominutum” (CMhm) [3] and “*Candidatus* Mycoplasma turicensis” (CMt) [4]. Another species, “*Candidatus* Mycoplasma haematoparvum-like”, has also been reported in cats [5–7]. Although these bacteria are distributed worldwide, the prevalence varies geographically [7–11].

It is still unknown how feline hemoplasmas are transmitted. Vector transmission through fleas [12–16] or ticks [17, 18] has been suggested, but direct transmission through aggressive interactions or blood transfusion have also been hypothesized as potential sources of infection.

Clinical presentation varies from absence of clinical signs to the existence of acute haemolytic anaemia, showing the affected cats pallor, depression, lethargy, weight loss, anorexia, dehydration and intermittent pyrexia or even sudden death [1]. In this sense, Mhf seems to be the most pathogenic of the three main feline hemoplasmas. The clinical presentation can vary depending not only on the pathogenicity of the haemoplasma species, but also on host factors, such as the presence of concurrent disease. Younger cats are more susceptible to clinical haemoplasmosis [19]. Other factors such as infecting organisms’ dose or route of infection may also impact on outcome [1].

There are just a few studies reporting hemoplasma infection in cats in Spain [9, 20, 21] and, to our knowledge, no epidemiological studies on these bacteria have been performed in the central region of the country. The objective of this study was to determine the prevalence of feline hemoplasmas (Mhf, CMhm and CMt) in cats from Madrid, central Spain, and to characterize risk factors and clinical signs associated with these feline infections in the area.

## Methods

### Recruitment and data collection

During a three-year period, blood samples were collected from 456 client-owned cats admitted to the Veterinary Medicine Teaching Hospital (VMTH) of the Complutense University of Madrid and 138 stray cats attended to be neutered/spayed in different clinics or animal protection societies located in the Community of Madrid. Client-owned cats from which blood samples were collected were not subjected to any other inclusion or exclusion criteria.

Data obtained at the time of enrolment of the client-owned cats in the study included signalment, environmental and lifestyle characteristics, such as living in urban/periurban/rural areas, travel history, and outdoor access; contact with other animals, exposure to arthropods and administration of ectoparasiticides; and clinical data, including previous tetracyclines treatment (in the last 60 days) or blood transfusions. Data collected from cats from animal protection societies were scarce, and included gender, living area and FeLV/FIV status. In addition, the date on which blood sample was drawn was recorded for all the cats included in this study.

Feline leukemia virus (FeLV) and feline immunodeficiency virus (FIV) status were tested using a commercial kit (IDEXX Laboratories, Inc., Westbrook, Maine, USA) and clinical signs and laboratory findings (erythrocyte, leukocyte and platelets count, haemoglobin, haematocrit) were also recorded. Feline infectious peritonitis (FIP) status was also checked using a commercial in-house antibody test (ImmunoComb FCoV Antibody Test Kit, Biogal, Kibbutz Galed, Israel) to detect seropositive feline coronavirus (FCoV).

### Nucleic acid extraction, quality control and PCR amplification

An UltraCleanTM DNA Blood Spin Kit (Mo Bio Laboratories, Carlsbad, USA) was employed for the DNA extraction from whole blood. Before the PCR amplification, DNA was evaluated for quality at 260/280 nm and quantified at 260/230 nm using a NanoDrop^TM^ spectrophotometer (Thermo Scientific, Waltham, USA).

A total of 594 samples of genomic DNA were processed, using the PCR-protocol [8] based on the amplification of a partial sequence of the *16S* rRNA gene of feline hemotrophic mycoplasmas. As previously described by Kamrani et al. [12], amplified products of 193 bp were identified as CMhm, and products of 170 bp as Mhf or CMt. Thus, a second PCR protocol [11] was carried out using specific CMt primers in all the previous PCR-positive samples.

The sequences of the primers employed, and PCR protocols are shown in Table 1. A total of 5 μl of genomic DNA was added to 12.5 μl Premix Ex Taq^TM^ (Conda, Madrid, Spain), 7.5 μl of deionized sterile water and 0.25 μl (100 μM) of the primers in a 25.5 μl final volume of the reaction mixture. The reactions were carried out in an automatic DNA thermal cycler MasterCycler ep Gradient (Eppendorf, Hamburg, Germany), including every time negative and positive controls. The PCR amplification products were visualized by ethidium bromide fluorescence after electrophoresis in a 3% agarose gel at 115 V for 30 min for the first PCR and 1.5% agarose gel at 90 V for the second one.

**Table 1:** Primers and protocols used for the amplification of feline hemoplasmas and housekeeping GAPDH gene control

Target gene	Product size (bp)	PCR primers (5′–3′)	PCR conditions
*16S* rRNA hemoplasmas [8]	170 / 193	F: ACGAAAGTCTGATGGAGCAATA	94 °C, 1 min; 45 cycles [94 °C, 1 min; 65 °C, 1 min; 72 °C, 30 s]; 72 °C, 10 min
		R: ACGCCCAATAAATCCGRATAAT	
*CMt* [11]	138	F: AGAGGCGAAGGCGAAAACT	95 °C, 2 min; 30 cycles [95 °C, 10 s; 58 °C, 30 s; 72 °C, 30 s]; 72 °C 5 min
		R: CTACAACGCCGAAACACAAA	
*GAPDH-Housekeeping* [22]	282	F: CCTTCATTGACCTCAACTACAT	95 °C, 1 min; 45 cycles [94 °C, 4 s; 57 °C, 4 s; 72 °C, 3 s]; 72 °C, 1 min
		R: CCAAAGTTGTCATGGATGACC	

*Abbreviations*: *CMt* “*Candidatus* Mycoplasma turicensis”, *GAPDH* gliceraldehide-3-phosphate dehydrogenase

Purification of amplified DNA fragments obtained for the first PCR was obtained using the Speed Clean-Up kit (Biotools, Madrid, Spain) and subsequently sequenced in an automated 3730 DNA Analyzer using a Big Dye Terminator 3.1 (Applied Biosystems, Foster City, USA) in Secugen S.L. (Madrid, Spain). Data obtained were compared with reference sequences deposited in GenBank, using the Basic Local Alignment Search Tool (BLAST).

Negative DNA samples were submitted to internal control PCR to evaluate both the presence of the amplifiable DNA and the absence of inhibitor factors [22].

### Statistical analysis

Statistical associations between results obtained by PCR and epidemiological, and hematological data were analyzed in the case of client-owned cats using the Chi-square test or Fisher’s exact test, where appropriate, and odds ratio (OR) with a 95% CI. It was possible to perform these statistical analyses for the whole feline sample (stray and client-owned cats) for data regarding gender, living area, months of sample collection and FeLV/FIV status. Other epidemiological information (related to signalment, environment, prophylaxis measures, clinical signs and laboratory findings) was only available for client-owned cats. Thus, statistical associations between PCR results and these data were assessed only for this group of client-owned cats. The significance level was established at *P* < 0.05. In order to exclude any possible confounding factors, logistic regression analysis with backward elimination was complementary performed with those variables that showed a statistical association with any hemoplasma infection. Analysis of the data was carried out with the support of “Departamento de Ayuda a la Investigación, Área de Informática y Comunicaciones”, Complutense University of Madrid, employing the statistical software SAS, 9.4 (SAS Institute, Cary, NC, USA).

## Results

Out of the 594 cats included in this study, 138 (23.2%) were stray cats and 456 (76.8%) were client-owned cats. It was not possible to obtain epidemiological and/or clinical data from every cat involved in the study, especially in stray animals. Table 2 shows the information available from the whole sample of cats included in this study (stray and client-owned cats). Data gathered from the population of the client-owned cats are shown in Tables 3 and 4. In addition, these tables show the prevalences for the different hemoplasma species and the results for the assessment of associations between positivity to the hemoplasma PCR and the rest of data.

**Table 2:** Comparison of prevalences of hemoplasma infection and epidemiological data in stray and client-owned cats

Variable	Total no. of cats (%)	Number of positive cats (%)						
		Any hemoplasma	Mhf	CMhm	CMt	Mhf + CMhm	CMt + CMhm	CMt + Mhf
Lifestyle	594	63 (10.6)	22 (3.7)	48 (8.1)	3 (0.5)	7 (1.2)	2 (0.3)	1 (0.2)
Client-owned	456 (76.8)	41 (9.0)	10 (2.2)	36 (7.9)	2 (0.4)	5 (1.1)	1 (0.2)	1 (0.2)
Stray	138 (23.2)	22 (15.9)*	12 (8.7)*	12 (8.7)	1 (0.7)	2 (1.4)	1 (0.7)	0
Months of sample collection	594							
Warm months	289 (48.6)	40 (13.8)*	15 (5.2)	30 (10.4)*	2 (0.7)	5 (1.7)	2 (0.7)	0
Cold months	305 (51.4)	23 (7.5)	7 (2.3)	18 (5.9)	1 (0.3)	2 (0.6)	0	1 (0.3)
Gender	540							
Male	260 (51.8)	42 (16.1)*	15 (5.8)*	32 (12.3)*	2 (0.8)	5 (1.9)	1 (0.4)	1 (0.4)
Female	280 (48.2)	12 (4.3)	4 (1.4)	8 (2.9)	0	0	0	0
Living area	394							
Urban	223 (56.6)	11 (4.9)	3 (1.3)	10 (4.5)	0	2 (0.9)	0	0
Periurban	99 (25.1)	10 (10.1)	2 (2.0)	9 (9.1)	1 (1.0)	1 (1.0)	1 (1.0)	0
Rural	72 (18.3)	8 (11.1)	2 (2.8)	6 (8.3)	1 (1.4)	0	0	1 (1.4)
FeLV	445							
Yes	32 (7.2)	8 (25.0)*	3 (9.4)	6 (18.7)*	0	1 (3.1)	0	0
No	413 (92.8)	38 (9.2)	16 (3.9)	27 (6.5)	2 (0.5)	5 (1.2)	1 (0.2)	1 (0.2)
FIV	447							
Yes	23 (5.2)	10 (43.5)*	5 (21.7) *	6 (26.1)*	1 (4.3)	1 (4.3)	0	1 (4.3)
No	424 (94.8)	37 (8.3)	14 (3.3)	28 (6.6)	0	5 (1.2)	0	0

*Abbreviations*: *CMhm* “*Candidatus* Mycoplasma haemominutum”, *Mhf Mycoplasma haemofelis*, *CMt* “*Candidatus* Mycoplasma turicensis”

*Statistically significant differences (*P* < 0.05)

**Table 3:** Distribution of feline hemoplasma infection in client-owned cats in accordance with different epidemiological data

Variable	Total no. of cats (%)	Number of positive cats (%)						
		Any hemoplasma	Mhf	CMhm	CMt	Mhf + CMhm	CMt + CMhm	CMt + Mhf
	456	41 (9.0)	10 (3.2)	36 (7.9)	2 (0.4)	5 (1.1)	1 (0.2)	1 (0.2)
Age	422							
Young (≤ 1-year-old)	85 (20.1)	2 (2.3)	2 (2.3)	2 (2.3)	0	2 (2.3)	0	0
Adult (> 1-year-old)	337 (79.9)	31 (9.2)*	6 (1.8)	27 (8.0)	1 (0.3)	2 (0.6)	0	1 (0.3)
Spayed/neutered	397							
Yes	247 (62.2)	22 (8.9)	4 (1.6)	20 (8.1)	0	2 (0.8)	0	0
No	150 (37.8)	9 (6.0)	3 (2.0)	8 (5.3)	1 (0.7)	2 (1.4)	0	1 (0.7)
Breed	424							
European	294 (69.3)	26 (8.8)	6 (2.0)	22 (7.5)	1 (0.3)	2 (0.7)	0	1 (0.3)
Non-European	130 (30.7)	9 (6.9)	3 (2.3)	8 (6.1)	0	2 (1.5)	0	0
Outdoor access	333							
Yes	82 (24.6)	10 (12.2)*	1 (1.2)	9 (11.0)	1 (1.2)	0	0	1 (1.2)
No	251 (75.4)	12 (4.8)	4 (1.6)	11 (4.4)	0	3 (1.2)	0	0
Contact with other animals	331							
Yes	225 (68.0)	18 (8.0)	2 (0.9)	17 (7.6)	1 (0.4)	1 (0.4)	0	1 (0.4)
No	106 (32.0)	8 (7.5)	3 (2.8)	7 (6.6)	0	2 (1.9)	0	0
Previous tick infestation	317							
Yes	16 (5.9)	0	0	0	0	0	0	0
No	301 (94.1)	21 (7.0)	5 (1.7)	19 (6.3)	1 (0.3)	3 (1.0)	0	1 (0.3)
Previous flea infestation	316							
Yes	41 (13.0)	4 (9.8)	0	4 (9.8)	0	0	0	0
No	275 (87.0)	17 (6.2)	5 (1.8)	15 (5.4)	1 (0.4)	3 (1.1)	0	1 (0.4)
Ectoparasiticide treatment	309							
Yes	86 (27.8)	8 (9.3)	2 (2.3)	7 (8.1)	0	1 (1.2)	0	0
No	223 (72.2)	13 (5.8)	3 (1.3)	12 (5.4)	1 (0.4)	2 (0.9)	0	1 (0.4)
Travel history	309							
Yes	116 (37.5)	7 (6.0)	0	7 (6.0)	0	0	0	0
No	193 (62.5)	14 (7.2)	5 (2.6)	12 (6.2)	1 (0.5)	3 (1.5)	0	1 (0.5)
Previous blood transfusion	314							
Yes	4 (1.3)	0	0	0	0	0	0	0
No	310 (98.7)	21 (6.8)	5 (1.6)	19 (6.1)	1 (0.3)	3 (1.0)	0	1 (0.3)
Tetracyclines treatment	314							
Yes	13 (4.1)	1 (7.7)	0	1 (7.7)	0	0	0	0
No	301 (95.9)	20 (6.6)	5 (1.7)	18 (6.0)	1 (0.3)	3 (1.0)	0	1 (0.3)

*Abbreviations*: *CMhm* “*Candidatus* Mycoplasma haemominutum”, *Mhf Mycoplasma haemofelis*, *CMt* “*Candidatus* Mycoplasma turicensis”

*Statistically significant differences (*P* < 0.05)

**Table 4:** Distribution of feline hemoplasma infection in client-owned cats in accordance with different haematological findings, and the presence or absence of clinical signs

Variable	Total no. of cats (%)	Number of positive cats (%)						
		Any hemoplasmas	Mhf	CMhm	CMt	Mhf + CMhm	CMt + CMhm	CMt + Mhf
	456	41 (9.0)	10 (3.2)	36 (7.9)	2 (0.4)	5 (1.1)	1 (0.2)	1 (0.2)
Clinical signs	425							
Yes	325 (76.5)	29 (8.9)	8 (2.5)	24 (7.4)	1 (0.3)	3 (0.9)	0	1 (0.3)
No	100 (23.5)	5 (5.0)	1 (1.0)	5 (5.0)	0	1 (1.0)	0	0
Coronavirus seropositivity	304							
Yes	10 (3.3)	1 (10.0)	1 (10.0)	1 (10.0)	0	1 (10.0)	0	0
No	294 (96.7)	24 (8.2)	6 (2.0)	21 (7.1)	1 (0.3)	3 (1.0)	0	1 (0.3)
Haematology								
RBC (× 10^6^ μl)	331							
High (> 10)	60 (18.1)	4 (6.7)	0	4 (6.7)	0	0	0	0
Normal (5–10)	251 (75.8)	23 (9.2)	3 (1.2)	21 (8.4)	2 (0.8)	1 (0.4)	1 (0.4)	1 (0.4)
Low (< 5)	20 (6.0)	6 (30.0)*	2 (10.0)*	5 (25)*	0	1 (5.0)	0	0
HGB (g/dl)	407							
High (> 15)	16 (3.9)	1 (6.2)	0	1 (6.2)	0	1 (6.2)	0	0
Normal (9–15)	332 (81.6)	26 (7.8)	6 (1.8)	23 (6.9)	2 (0.6)	3 (0.9)	0	1 (0.3)
Low (< 9)	59 (14.5)	12 (20.3)*	2 (3.4)	11 (18.6)*	0	0	1 (1.7)	0
Haematocrit (%)	410							
High (> 45)	22 (5.4)	1 (4.5)	0	1 (4.5)	0	0	0	0
Normal (24–45)	360 (87.8)	32 (8.9)	6 (1.7)	29 (8.1)	2 (0.6)	3 (0.8)	1 (0.3)	1 (0.3)
Low (< 24)	28 (6.8)	6 (21.4)*	2 (7.1)	5 (17.9)	0	1 (3.6)	0	0
MCV (fl)	333							
High (> 55)	5 (1.5)	2 (40.0)	1 (20.0)	1 (20.0)	1 (20.0)	0	1 (20.0)	0
Normal (39–55)	252 (75.7)	25 (9.9)	4 (1.6)	23 (9.1)	1 (0.4)	2 (0.8)	0	1 (0.4)
Low (< 39)	76 (22.8)	4 (7.9)	0	6 (7.9)	0	0	0	0
MCH (pg)	335							
High (> 17.5)	7 (2.1)	2 (28.6)	1 (12.5)	1 (14.3)	1 (12.5)	0	1 (12.5)	0
Normal (12.5–17.5)	273 (81.5)	27 (9.9)	3 (1.1)	25 (9.2)	1 (0.4)	1 (0.4)	0	1 (0.4)
Low (< 12.5)	55 (16.4)	4 (7.3)	1 (1.8)	4 (7.3)	0	1 (1.8)	0	0
MCHC (g/dl)	405							
High (> 36)	10 (2.5)	2 (20.0)	1 (10.0)	1 (10.0)	1 (10.0)	0	0	1 (10.0)
Normal (30–36)	381 (94.1)	36 (9.4)	6 (1.6)	34 (8.9)	1 (0.3)	4 (1.0)	1 (0.3)	0
Low (< 30)	14 (3.5)	1 (7.1)	1 (7.1)	0	0	0	0	0
Leukocytes (× 10^3^ μl)	408							
High (> 14)	77 (18.9)	5 (6.5)	1 (1.3)	5 (6.5)	0	1 (1.3)	0	0
Normal (5.5–14)	273 (66.9)	29 (10.6)	7 (2.6)	25 (9.2)	2 (0.7)	3 (1.1)	1 (0.4)	1 (0.4)
Low (< 5.5)	58 (14.2)	5 (8.6)	0	5 (8.6)	0	0	0	0
Platelets (× 10^3^ μl)	148							
High (> 800)	12 (8.1)	0	0	0	0	0	0	0
Normal (800–300)	60 (40.5)	6 (10.0)	3 (5.0)	5 (8.3)	0	2 (3.3)	0	0
Low (< 300)	76 (51.3)	7 (9.2)	1 (1.3)	7 (9.2)	0	1 (1.3)	0	0

Abbreviations: *CMhm* “*Candidatus* Mycoplasma haemominutum”, *Mhf Mycoplasma haemofelis*, *CMt* “*Candidatus* Mycoplasma turicensis”, *RBC* red blood cell count, *HGB* haemoglobin concentration, *MCV* mean corpuscular haemoglobin, *MCH* mean corpuscular haemoglobin, *MCHC* mean corpuscular haemoglobin concentration

*Statistically significant differences (*P* < 0.05)

The overall prevalence of hemoplasma infection in cats from Madrid was 10.6% (63/594). The prevalences of CMhm, Mhf and CMt were 8.1% (48/594), 3.7% (22/594) and 0.5% (3/594), respectively. A total of seven cats (1.17%) were co-infected with CMhm and Mhf, two (0.33%) with CMhm and CMt and another one (0.17%) with Mhf and CMt. No single infection with CMt was detected.

Attending to the different lifestyle groups, prevalence of hemoplasma infection in stray cats was 15.9% (22/138) and prevalence in client-owned cats was 9% (41/456), the lifestyle (stray) of the cat being statistically associated with hemoplasma infection (*χ*^2^ = 5.40, *df* = 1, *P* = 0.020). The same was observed when attending to Mhf infection (*χ*^2^ = 12.56, *df =* 1*, P =* 0.0004), with 8.7% (12/138) positive to Mhf in stray cats, and 2.2% (10/456) in client-owned cats, but not in the case of CMhm or CMt. Data related to prevalence in different lifestyle groups are shown in Table 2.

Considering any of the hemoplasma infections assessed in stray and client-owned cats of this study, the season of the sample collection was significantly associated with positivity by PCR. Specifically, 63.5% (40/63) of the positive samples for any of the hemoplasmas were collected during spring and summer (*χ*^2^ = 6.21, *df =* 1, *P =* 0.013). When considering this association in the different species of the study separately, the same was observed for CMhm (*χ*^2^ = 4.01, *df* = 1, *P =* 0.045), with 62.5% (30/48) samples drawn in spring or summer, while Mhf infection was detected more frequently specifically in the samples collected during summer months in comparison with the other seasons together (*P =* 0.032, OR = 0.35; 95% CI: 0.13–0.89).

Within other epidemiological data available from stray and client-owned cats of the study, significant associations were detected between male gender and CMhm (*χ*^2^ = 17.55, *df =* 1*, P* < 0.0001), Mhf (*χ*^2^ = 7.48, *df =* 1, *P =* 0.006) and overall hemoplasma infection (*χ*^2^ = 21.10, *df =* 1, *P <* 0.0001).

When analyzing epidemiological information that was available from client-owned cats only, some additional statistically significant associations between these data and hemoplasma prevalence were detected. Client-owned cats older than one year of age showed a significantly greater risk for hemoplasma infection (*χ*^2^ = 4.41, *df =* 1, *P =* 0.036). This finding was not found when considering CMhm (*χ*^2^ = 3.40, *df* = 1, *P =* 0.065), or Mhf (*P =* 0.665, OR = 0.75; 95% CI:0.15–3.79) infection alone.

Having an outdoor access was identified as an additional risk factor for any hemoplasma species in client-owned cats (*χ*^2^ = 5.51, *df* = 1, *P =* 0.019). None of the other epidemiological data evaluated were associated with hemoplasma infection.

Retroviral status was confirmed as a risk factor in stray and client-owned cats. Cats positive for FeLV were more likely to be infected with any hemoplasma (*P =* 0.011, OR = 8.04; 95% CI: 3.3–19.6) and with CMhm (*P =* 0.023, OR = 3.3; 95% CI: 1.25–8.7). In a similar way, FIV-infected cats exhibited higher prevalences of CMhm (*P =* 0.005, OR = 4.99; 95% CI: 1.82–13.65), Mhf (*P =* 0.002, OR = 8.13; 95% CI: 2.64–25.06) and overall hemoplasma infection (*P <* 0.0001, OR = 8.04; 95% CI: 3.3–19.61).

No statistically significant differences in hemoplasmas’ prevalences were detected between symptomatic and asymptomatic client-owned cats (Table 4). However, when specific clinical signs were evaluated, muscle-skeletal (15.8%, 6/38) and renal (11.2%, 13/116) signs were associated with CMhm infection (*P =* 0.035, OR = 2.97; 95% CI: 1.13–7.82, and *χ*^2^ = 4.82, *df =* 1, *P =* 0.028*,* respectively).

Hemoplasma-infected cats showed some remarkable findings in the hematological analysis. Low red blood cell (RBC) count was associated with infection by any hemoplasma (*P =* 0.009, OR = 0.22; 95% CI: 0.07–0.62), and specifically with Mhf (*P =* 0.031, OR = 0.09; 95% CI: 0.01–0.56) and CMhm (*P =* 0.025, OR = 0.26; 95% CI: 0.08–0.78) infection*.* Low haemoglobin (HGB) concentration was significantly associated with any hemoplasma and CMhm infection (*χ*^2^ = 9.21, *df =* 1, *P =* 0.002 and *χ*^2^ = 8.86 *df =* 1, *P =* 0.003, respectively) and low haematocrit (HTC) was associated with any hemoplasma infection (*P =* 0.004, OR = 0.34; 95% CI: 0.13–0.91). These results are shown in Table 4.

Ten cats were seropositive to FIP (3.29%, 10/304), with also one of these animals co-infected with CMhm and Mhf (*P* = 0.582, OR = 1.25; 95% CI: 0.15–10.29), but PCR for FIP was not performed. This animal was a young non-neutered male client-owned cat that was attended at the VMTH with musculoskeletal and gastrointestinal signs.

Data available for the two client-owned cats with CMt infection are also detailed in Tables 2, 3 and 4. One of them was co-infected with Mhf and the other with CMhm*.* The one co-infected with *Mhf* was a non-neutered adult male (3 years old), client-owned cat with outdoor access, living in a rural area and positive for FIV. It was found to have a slight monocytosis, without other abnormalities in blood analysis. No previous contact with ectoparasites was reported. Unfortunately, the data available for the client-owned cat co-infected with CMt and CMhm was very scarce. Finally, another co-infected cat with CMhm and CMt was a 15 year old male stray cat, negative for retrovirus and with a high total protein concentration (9.7 g/dl).

## Discussion

The presence of hemotropic mycoplasma infections in cats from central Spain has been demonstrated in this study, with CMhm being the most prevalent species*.* The overall prevalence of hemoplasma infection in cats in the Madrid area determined in the current study (10.6%; 63/594) is comparable to the results of a previous study carried out in Barcelona, Spain [20], where 12% of a population of 191 cats with and without outdoor access was analysed. This hemotropic mycoplasma prevalence is also similar to those described in other studies performed in Denmark [23], Germany [24, 25], Italy [26] and Switzerland [10] and lower than the rates reported by others in Greece [27], Italy [28, 29] and Portugal [6, 30]. These differences in prevalence can be due to several factors: the cat population sampled (which could present several risk factors simultaneously), geographical variations and/or differences in the diagnostic technique used in these studies (from microscopic to molecular detection among others). Our study was performed employing conventional PCR that has been highly employed previously and with good sensitivity, but it should be considered that a higher sensitivity could be achieved with a combination of real-time and conventional PCR analyses [5, 31, 32].

In agreement with our results, CMhm has been the most common hemoplasma found in the majority of prevalence studies carried out worldwide. This could be due, as suggested by Tanahara et al. [11], to a more efficient infection and multiplication of CMhm in comparison to other hemotropic mycoplasma, or to a lower virulence that allows a longer asymptomatic carrier state.

The presence of “*Candidatus* Mycoplasma haematoparvum” DNA has not been evaluated in this study. Considering the description of this infection in other countries [5–7], future work should be done to analyse this hemotropic mycoplasma species in central Spain.

Consistent with previous observations [6, 33, 34] of a higher prevalence of feline hemoplasma infection in countries with warmer climates, when analysing season of collection of blood samples in the current study, a statistically significant higher prevalence of hemoplasma infection during spring and summer was found, with most positive cats (63.5% of positive samples, 40/63) included in the study between April and September. This seasonal influence on prevalence has been previously described when comparing summer with autumn in one study performed in Italy [28], suggesting vector transmission. However, it has not been proven for natural infection and other studies have not found any association between prevalence rate and season of the year [6, 28, 33, 34]. In fact, in the client-owned cat group of this study, history of previous ectoparasites infestation (ticks/fleas) was not found associated with hemoplasma infection, similarly to previous descriptions [6, 33]. However, this information must be carefully assessed considering the natural behaviour of grooming in the cat, that could have facilitated the fact that arthropod infestation went unnoticed for the owner/person in charge of the cat [28]. In addition, other means of transmission must exist, considering their detection in areas where there is absence of the possible vectors [1]. In this sense, it has been described that male cats are more likely to engage in roaming and fighting behaviour, which may increase their chance of contracting the disease if a direct mode of transmission exists [7, 11, 20, 26, 33, 35–41]. Our own study supports a higher risk for male animals and for stray cats or client-owned cats that had outdoor access. All these factors may favour contact with other cats, although it should be considered that stray or client-owned cats with outdoor access are supposed to be more exposed not only to contact with other cats, but also to ectoparasites. In addition, it is important to consider the results of the logistic regression study carried out, that showed a stronger relationship of mycoplasma infection with the variable FIV status, which could mean that gender variable could be a confounding factor due to the relationship between positive FIV status and male sex.

The significant association between hemoplasma infection and the co-infection with retrovirus (FeLV/FIV) shown by this and previous studies [6, 19, 25, 35, 39] could be explained by the well-known immunosuppressive effect of these retroviruses. However, since FIV is mainly transmitted through bite wounds, it has been suggested that the strong statistical association between this retroviral infection and feline hemoplasmosis could be supporting the horizontal route of transmission [20].

It has been frequently reported that there is an association between the age of the cats and hemoplasma infection. Some studies have described that adult age could be a risk factor for these infections [7, 11, 23, 26, 33, 35, 40, 41]. In our study, being an adult cat (> one year-old) was statistically associated with hemoplasma infection. This association could be explained because older animals have been more exposed to blood-sucking arthropods and to more aggressive interactions with other cats. Moreover, this association between hemoplasma infection and adult cats could be explained by the existence of carrier cats with a chronic infection as described previously [42].

Clinical signs and laboratory findings detected for hemoplasma infection depend on a wide range of factors. Concurrent diseases or previous infections, hemoplasma species involved (CMhm, Mhf, CMt or even co-infections), and the stage (acuteness or chronicity) of hemoplasma infection could considerably change these findings. While the most frequently described clinical signs in cats with hemoplasmosis are related to the occurrence of anaemia, lethargy, or pale mucous membranes [1] among others, the current study showed statistical associations between the infection by CMhm and musculoskeletal and renal signs. These results should be interpreted with caution, considering that other diseases could be responsible for the clinical signs detected in the cats in the current study, as it was observed in the cat co-infected with FIP, CMhm and Mhf, that showed musculoskeletal and gastrointestinal signs. Sykes et al. [41] suggested a relation between renal signs and hemoplasma infection, but also considered that the infected population represented by old cats probably has a high prevalence of chronic kidney disease.

On the other hand, our study showed some remarkable laboratorial findings, such as low HTC, RBC count and HGB concentration. This and other studies [27] have detected hemoplasma infection both in cats suffering anaemia and in cats with red blood cells counts within reference values. Mhf is considered the most pathogenic feline hemoplasma species, causing extravascular erythrophagocytosis especially during the acute phase. However, an interesting finding to highlight is the association between the existence of anaemia not only in Mhf, but also in CMhm infected cats. It has been previously described that experimental CMhm infection generally does not induce anaemia and significant clinical signs, so geographical differences in the pathogenicity of the strain of this species should be considered. Nevertheless, it is important to note that the cats of the study could have been exposed to other pathogens previously described in central Spain [43, 44] or could be affected by other diseases associated with the development of anaemia.

This study also showed the presence of CMt in central Spain, although the low number of positive cats precludes a proper epidemiological analysis for this species.

## Conclusions

To our knowledge, this is the first epidemiological survey of feline hemoplasmas performed in central Spain (Madrid). Our study confirms that the three-main species of hemoplasma are infecting client-owned and stray cats in this region of Spain, with “*Ca.* Mycoplasma haemominutum” being the most prevalent species. Male gender, collection of blood during warm months, FeLV/FIV positivity status, and outdoor access were associated with hemotropic mycoplasma infection in cats from Madrid. With regard to clinical signs and laboratory parameters, natural infection by feline hemoplasmas in the area is associated with anaemia, being difficult to determinate if co-infections can influence this finding. More studies are necessary to understand the role of the infection by these species of hemoplasma in cats.

## Abbreviations

CI: Confidence interval
CMhm: “*Candidatus* Mycoplasma haemominutum”
CMt: “*Candidatus* Mycoplasma turicensis”
FCoV: Feline coronavirus
FeLV: Feline leukemia virus
FIP: Feline infectious peritonitis
FIV: Feline immunodeficiency virus
GAPDH: Gliceraldehide-3-phosphate dehydrogenase
HGB: Haemoglobin concentration
Mhf: *Mycoplasma* *haemofelis*
MCH: Mean corpuscular haemoglobin
MCHC: Mean corpuscular haemoglobin concentration
MCV: Mean corpuscular haemoglobin
OR: Odds ratio
PCR: Polymerase chain reaction
RBC: Red blood cell count
RNA: Ribonucleic acid
VMTH: Veterinary Medicine Teaching Hospital
